# Ecological Study on Hospitalizations for Cancer, Cardiovascular, and Respiratory Diseases in the Industrial Area of Etang-de-Berre in the South of France

**DOI:** 10.1155/2013/328737

**Published:** 2013-06-20

**Authors:** Laurence Pascal, Mathilde Pascal, Morgane Stempfelet, Sarah Goria, Christophe Declercq

**Affiliations:** ^1^French Institute for Public Health Surveillance, 12 rue du Val d'Osne, 94415 Saint-Maurice, France; ^2^South Regional Office of French Institute for Public Health Surveillance (Cire Sud), 132 boulevard de Paris, 13331 Marseille Cedex 3, France

## Abstract

The Etang-de-Berre area is a large industrialized area in the South of France, exposing 300,000 inhabitants to the plumes of its industries. The possible associated health risks are of the highest concern to the population, who asked for studies investigating their health status. A geographical ecological study based on standardized hospitalizations ratios for cancer, cardiovascular, and respiratory diseases was carried out over the 2004–2007 period. Exposure to air pollution was assessed using dispersion models coupled with a geographic information system to estimate an annual mean concentration of sulfur dioxide (SO_2_) for each district. Results showed an excess risk of hospitalization for myocardial infarction in women living in districts with medium or high SO_2_ exposure, respectively, 38% [CI 95% 4 : 83] and 54% [14 : 110] greater than women living in districts at the reference level exposure. A 26% [2 : 57] excess risk of hospitalization for myocardial infarction was also observed in men living in districts with high SO_2_ levels. No excess risk of hospitalization for respiratory diseases or for cancer was observed, except for acute leukemia in men only. Results illustrate the impact of industrial air pollution on the cardiovascular system and call for an improvement of the air quality in the area.

## 1. Introduction

Relationships between urban air pollution and hospitalizations for cardiorespiratory causes are well established in many studies around the world [1–3] and in France [4]. By comparison, published studies about the health effects of industrial air pollution on population living near industries are sparse, and few studies investigate the impact of industrial air pollution on cardiovascular or respiratory hospitalizations [5–7]. This paper presents the first study on the impacts of industrial air pollution on cardiorespiratory hospitalizations, in one of the largest industrial areas in France.

The Etang-de-Berre area is a large pond (0.15 km^2^) surrounded by three major industrial complexes gathering several oil refineries, chemical plants, ironworks, metal plants, a waste incineration plant, an airport, and the largest French seaport [8, 9]. This industrial area located in the Provence-Alpes-Côte d'Azur region has experienced a strong economic growth since the 70s. The population has doubled between 1970 and 2000, and, today, about 300,000 inhabitants are more or less exposed to the plumes of industries. 

The contribution of the Etang-de-Berre area to the regional emissions is estimated at 58% for sulfur dioxide (SO_2_), 13% for particulate matter under 10 *μ*m (PM_10_), 23% for nitrogen oxide (NO_*x*_), and 10% for volatile organic compounds (VOC). The main sources are the industries and and the production of energy for SO_2_ and VOC emissions, industries and road traffic for PM_10_ emissions, and industries, production of energy, and road traffic for NO_*x*_ emissions [10]. 

SO_2_ concentrations measured by the Air Quality Network in this area are still the highest observed at the regional level, even if they had decreased regularly during the last 20 years. In 2008, all monitoring stations in the area exceeded the 2005 World Health Organization (WHO) Air quality guidelines for maximum daily mean concentrations (20 *μ*g·m^−3^). None exceeded the European Council Directive 2008/50/EC of 21 May 2008 hourly limit values (hourly mean >350 *μ*g·m^−3^/more than one day) [11]. PM_10_ concentrations are relatively stable 10 years ago, but some peaks are still measured. In 2008, all the monitoring stations exceeded the WHO air quality guidelines (annual mean of 20 *μ*g·m^−3^). None exceeded the 2008/50/EC limit value (annual mean of 40 *μ*g·m^−3^). Nitrogen oxides (NO_*x*_), heavy metals, and polycyclic aromatic hydrocarbons (PAH) concentrations were under the 2008/50/EC limit value, whereas benzene concentrations were slightly higher near the industrial sites. Ozone concentrations were high in summer because of the emissions of ozone precursors and the high degree of sunshine but this affects all the regional area.

Since the 1990s, environmental protection associations created by the population request an assessment of the health of population living near these polluting and potentially dangerous industries.

The administrative authorities decided to carry out quantitative health risk assessments (HRA), based on the comparison of exposure to pollutants with toxicological reference values (TRV), for the three main industrial complexes between 2006 and 2011. 

The first HRA, on the oil refining area of Berre-l'Etang, began in 2006 and revealed high benzene and 1.3 butadiene fugitive emissions at the refinery [12]. Carcinogenic risks by inhalation exposure were found above the reference threshold of 10^−5^ for the population living in the city of Berre-l'Etang and in a large northern part of the study area.

Corrective measures to reduce emissions of these two compounds were then implemented on the industrial site. An updated HRA carried out in 2008 showed a decrease of the area exposed to benzene, from 30 km^2^ to 10 km^2^ around the industrial site. Yet, carcinogenic risks by inhalation exposure were still above the reference threshold of 10^−5^ for the population living in the north part of the study area.

An HRA on the industrial-port area of Fos-sur-Mer [13] found that SO_2_ and PM_10_ modeled concentrations were higher than the air quality guidelines in all the study area. Chrome VI and 1,2-dichloroethane modeled concentrations were too high on the industrial site only. Carcinogenic risks by inhalation exposure were under the reference threshold of 10^−5^ for the entire population living near the industrial site. 

The last HRA on the petrochemical area of Lavéra-La Mède [14] found that SO_2_ and PM_10_ modeled concentrations were higher than the air quality guidelines in all the study area. Benzene levels were too high and dangerous for workers on the industrial site only. Carcinogenic risks by inhalation exposure were above the limit threshold of 10^−5^ for the population living in a part of the study area representing 21,000 inhabitants.

These studies have led to a complete inventory of the different pollutants emitted by the industries and have helped prioritizing actions to reduce the exposure of the population. SO_2_ and PM_10_ pollutants were classified as requiring priority actions to reduce industrial emissions and population exposure, although it was not possible to assess the related health risks in the HRA, as TVR are not available for these compounds. Decreasing benzene, 1,3-butadiene, chrome VI, and 1,2-dichloroethane industrial emissions was also recommended to decrease the exposure of workers and of the population neighboring the industrial sites.

However, these studies cannot answer the main concern of the population: is the health of the people living in this industrial area worse than the health of people living in nonindustrial areas? 

Therefore, the administrative authorities asked the Regional office of the French Institute for Public Health Surveillance to carry out an epidemiological study. After a review of the existing studies and of the routinely available data for this area, we decided to conduct an ecological study on hospitalizations data. The objective of this ecological study was to estimate a relationship between hospitalizations ratios and SO_2_ exposure levels at the district of residence. Comparison was done between exposed and nonexposed district, controlling on socioeconomic status estimated through Townsend's index and proportion of male workers in each district, which are factors potentially influencing people health and exposure. 

## 2. Materials and Methods

### 2.1. Study Area

**Figure 1:**
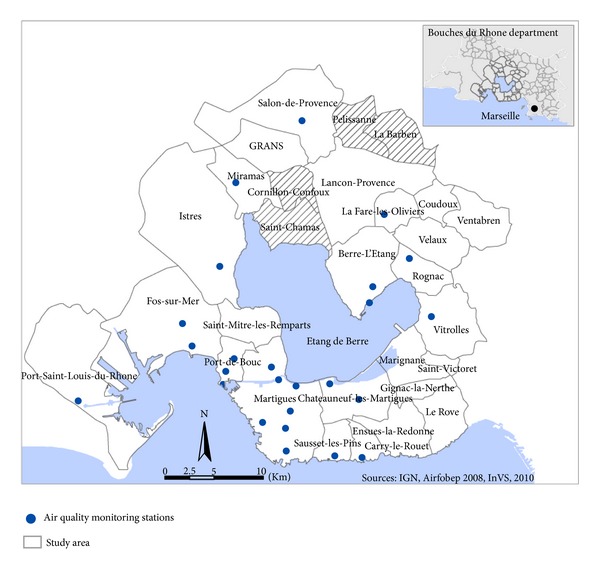
Study area and localization of air quality monitoring stations.

The study area is located in the Provence-Alpes-Côte-d'Azur region near the Mediterranean Sea. Its boundaries were based on modeled SO_2_ concentrations, topographic criteria, and labour pool. It included 29 administrative districts (named districts afterwards) surrounding the Etang-de-Berre pond and represented 399,962 inhabitants living on a 975 km^2^ area (Figure 1). 430 plants classified for environmental protection are located in the study area. Almost 50 of them have dangerous activities related to a high risk of industrial accident and are classified as “high threshold” according to the European Council Directive 96/82/EC of 9 December 1996 on the control of major-accident hazards involving dangerous substances.

**Figure 2:**
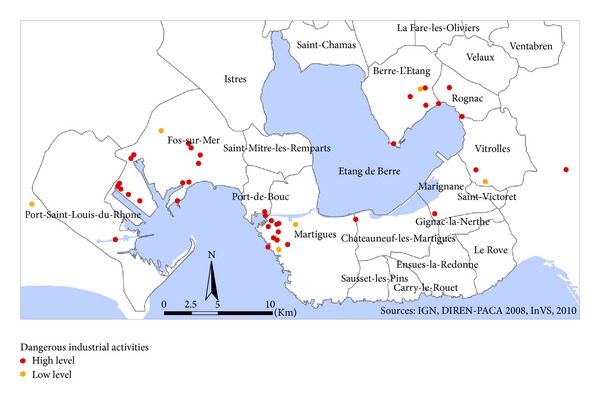
The industrial surrounding of Etang-de-Berre area. High and low level refers to the European Council Directive 96/82/EC of 9 December 1996 on the control of major-accident hazards involving dangerous substances.

These industries are grouped in 3 main complexes (Figure 2):

- the Lavera-la Mède area located in the district of Martigues, operating oil refining, petrochemical and organic chemical activities, and chlorine chemistry since the 1950s;
- the Berre area located in the district of Berre-l'Etang operating oil storage and petrochemical industry. The first refinery was settled in 1933;
- the industrial port area of Fos-sur-Mer including steel and metal working, chemicals plants, waste incineration plant, and the port for ore and oil tankers settled since the 1970s.

The Etang-de-Berre area is also crossed by a dense road network which supports a high traffic of heavy trucks related to the industrial and harbor facilities and of passenger cars commuting from home to work. 

### 2.2. Exposure Assessment

The local Air Quality Network (Air PACA) measures air pollution levels since 1972. In 2008, 27 monitoring stations settled on the study area (Figure 1) measured continuously the following pollutants: sulfur dioxide (SO_2_), ozone (O_3_), nitrogen dioxide (NO_2_), particulate matter (PM_10_), carbon monoxide (CO), and benzene. For SO_2_ concentrations in the study area, annual mean levels of the different monitoring stations varied between 2 and 18 *μ*g m^−3^ and maximum hourly mean levels between 83 and 831 *μ*g·m^−3^ (Table 1). The highest values are measured by the industrial monitoring stations. In comparison, for the 6 stations located on the rest of the region, annual mean levels varied between 1 and 4 *μ*g m^−3^ and maximum hourly mean levels between 20 and 132 *μ*g·m^−3^. 

**Figure 3:**
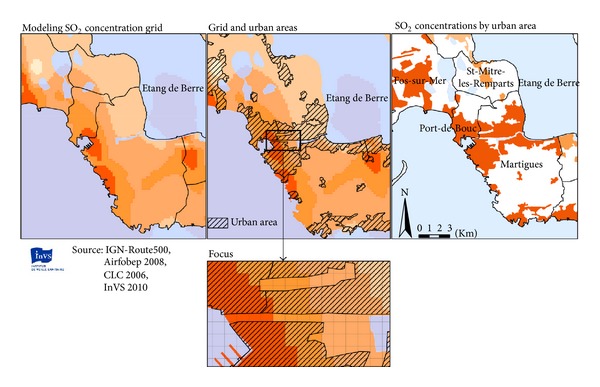
From SO_2_ concentrations grid to urban exposure estimation.

Exposure to air pollution was assessed at the district level, using SO_2_ concentrations as a proxy for industrial emissions. Air PACA provided the mean concentrations of SO_2_ for 2008 on a 200 m∗200 m grid using a dispersion model (ADMS4), a meteorological model and kriging. We used a geographic information system (GIS) to assign a concentration level to each district. To aggregate concentrations data, urban areas of each district were identified based on the 2006 land cover classification system. Urban areas included urbanized areas, major roads and railways, commercial, industrial, and working areas, leisure activities areas, and public gardens. For each district, the concentrations were averaged weighted on the cells proportion included in urban areas as illustrated in Figure 3.

**Figure 4:**
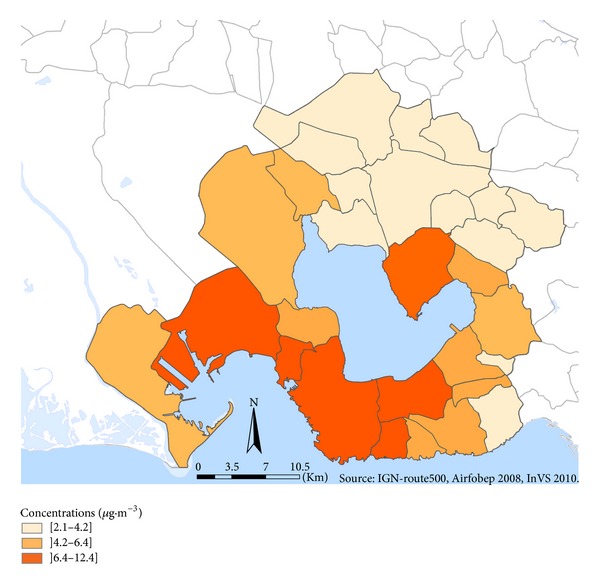
SO_2_ exposure estimations by district (2008 data).

The annual mean levels of SO_2_ varied between 2.1 and 12.4 *μ*g·m^−3^ depending on the district (Figure 4) and were grouped in three classes of exposure based on quartiles: reference (<4.2 *μ*g·m^−3^); medium (between 4.2 and 6.4 *μ*g·m^−3^), and high (>6.4 *μ*g·m^−3^) values (Table 2). Reference levels were similar to the SO_2_ annual mean levels measured in nonindustrial districts in the regional area, varying from 1 to 4 *μ*g·m^−3^. 

We also investigated whether PM_10_ concentrations could be an industrial pollution indicator for this ecological study. Annual mean levels of the different monitoring stations varied between 27 and 33 *μ*g·m^−3^ in the study area and were similar to those measured in the rest of the region (Table 3). With the same method used for SO_2_, the estimated annual mean levels of PM_10_ varied between 27.8 and 33.6 *μ*g·m^−3^ depending on the district. The spatial distribution of concentrations was relatively homogenous and the highest concentrations were not observed at industrial districts.

### 2.3. Hospitalization Data

The French programme for hospital information system (PMSI) is implemented since 1994 in public hospitals and since 1997 in private hospitals. A complete hospitalization database is available since 1998. It is a medical economic database based on the diagnosis-related group (DRG) method. Each hospitalization is registered in a local database grouped in a national database. Since 2004, a patient identification number is included to identify patients and hospital stays related to each patient. 

The national hospitalization database held by the PMSI provided hospitalization data for the whole region. Hospital stays included in the analysis were selected over the study period 2004–2007 based on several selection criteria. On the first step, we excluded stays without patient identification number and stays for patient that moved outside or inside the study area between 2004 and 2007. On the second step, stays for the studied diseases were selected from the main diagnosis at the discharge, coded with the 10th revision of the International Classification of Diseases (ICD-10), and sometimes from secondary diagnosis. Finally, patients living in the study area were selected from their zip codes. The first hospitalization of each resident over the study period was retained in order to approximate a hospitalization incidence for each health indicator.

Respiratory and cardiovascular hospitalization indicators have been selected from the papers on links between air pollution and health [15–27]. The selection of cancer hospitalization indicators was based on knowledge about frequencies of different type of cancer at the regional level and on the results of two French reports on environmental cancers [28, 29]. The following hospitalization indicators were defined:

- all cardiovascular diseases (ICD-10: I00–I99), heart diseases (ICD-10: I00–I52), and coronary heart diseases (ICD-10: I20–I24), myocardial infarction (ICD-10: I21-I22), stroke (ICD-10: I60–I64 or G45-G46), heart rate disorders (ICD-10: I44–I49), coronary heart diseases with heart rate disorders (ICD-10: I20–I24 as main diagnosis and I44–I49 as secondary diagnosis);
- all respiratory diseases (ICD-10: J00–J99), respiratory infections (ICD-10: J04–J06 or J10–J18 or J20–J22), pneumonia (ICD-10: J10–J18), asthma (ICD10: J45-J46), and exacerbations of chronic obstructive pulmonary diseases (principal indicator algorithm described in [30]);
- all cancers (ICD10: C00–C97), lung cancer (ICD10: C33-C34), bladder cancer (ICD10: C97), breast cancer (ICD10: C50), multiple myeloma (ICD10: C90), malignant non-Hodgkin's lymphoma (ICD10: C82–C85), and acute leukemia (ICD10: C910, C920, C924, C925, C930, C942, C943, C950).

The number of hospitalizations selected for the study area represented 9% of the cardiovascular and respiratory diseases hospitalizations and 7% for cancers hospitalizations registered at the regional level.

### 2.4. Confounding Factors

The 2006 national census held by the French national institute for statistics and economic studies (INSEE) provided data on socio-occupational groups of the working population in the study area and for the socioeconomic items included in Townsend's index. This index was built using the following socioeconomic items: proportion of unemployed person among working population, proportion of main homes with more than one person per room, proportion of main homes occupied by not owner household, and proportion of household without a car [31]. Standardized socioeconomic variables using regional values as reference were used to build an additive scale for each district. 

The proportion of male workers was retained as a confounding factor, making the hypothesis that it would be a good predictor of the industrialization of each district.

### 2.5. Statistical Analysis

We performed a descriptive analysis of the exposure, socioeconomic, and hospitalizations data. We calculated the expected number of cases at the district level for each health indicator by standardization method using the regional population as reference. Then standardized hospitalization ratios (SHR) were calculated as the rate of observed to expected cases. 

Relative risks of hospitalization for people living in medium or high exposed districts were calculated compared to those living in the reference districts. Overdispersed Poisson regression models were fitted to assess the association between hospitalization ratios and classes of exposure to industrial pollution, taking into account potential confounding factors. The Bayesian hierarchical model developed by Besag et al. (BYM) [32] was fitted to account for this extra Poisson variability.

The first level of the BYM is a classical Poisson regression model. The second level splits the residual risk into a linear combination of covariate effects *xTiβ* and into random effects *U*_*i*_ and *V*_*i*_ measuring excess heterogeneity and spatial similarity, respectively:

(1) $$\begin{array}{c} \text{Log}\left(\theta_{i}\right)=\alpha +xTi\beta +U_{i}+V_{i}, \end{array}$$

where the term exp⁡⁡(*α*)  is the overall relative risks of disease in the study area compared to the reference rate.

The vectors *U* and *V* are supposed independent, and *U*, that models the excess heterogeneity of the relative risks, is assumed to follow a normal distribution *U*_*i*_ ~ *N*  (0, *σ*_*u*_^2^). To model spatial similarity in residuals the Gaussian conditional autoregressive model (CAR) is used as the prior for the spatial component *v*:

(2) $$\begin{array}{c} \left(\frac{V_{i}}{V_{j}}=\;\nu_{i},\;\;j\neq i\right)\sim N\left(\frac{\sum_{j\neq i}^{}{w_{ij}v}_{j}}{\sum_{j\neq i}^{}w_{ij}},\frac{\sigma_{v}^{2}}{\sum_{j\neq i}^{}w_{ij}}\right), \end{array}$$

where the *w*_*ij*_s denote weights defining which districts *j* are neighbors to district *i* (by convention *w*_*ii*_ = 0 for all *i*). We used the adjacency-based weights where *w*_*ij*_ = 1 if district *j* is adjacent to district *i*, *w*_*ij*_ = 0 otherwise are used. We have taken Gamma prior distributions for the precision parameters (reciprocal of the variance) of the heterogeneity and spatial terms. For both we have taken the noninformative Γ (0.5, 0.0005). The Γ(*a*, *b*) denotes the Gamma distribution with expectation equal to *a*/*b*. Non-informative priors were taken for the other parameters, that is, the intercept and the regression coefficients.

In a Bayesian context, we defined the credible interval at the 5%, that is, the probability that the parameter belongs to is 95%. Analysis was done by age (children 0–14 years, adults over 15 years) and by sex for the adults with the software R and WinBUGS. 

## 3. Results

The highest SO_2_ levels (>6.4 *μ*g·m^−3^) were observed in the highly industrialized districts in the South of the Etang-de-Berre area. Districts in the Northeast of the study area had the lowest levels of air pollution (Figure 3).

The Townsend's index values ranged from −3.5 to 7.9. High values are related to a low socioeconomic status (SES) and negative values to a rather high SES. Districts in the North of the study area were rather favored and industrial districts rather deprived. This index was significantly correlated with the socio-occupational group but moderately with SO_2_ exposure levels (coefficient of correlation = 0.4).

Table 4 presents the number of cases by hospitalization indicators for the whole population. Cardiovascular diseases were the main causes of hospitalizations. For all indicators, the number of cases varied between districts depending on the population size. 

The number of cases varied also according to sex and age. The sex ratio male/female varied from 1.2 for all cardiovascular diseases to 2.4 for myocardial infarction (MI) hospitalizations. Hospitalizations for exacerbations of COPD occurred rather in males (sex ratio = 2.5) while hospitalizations for respiratory infections, pneumonia, or asthma occurred in both sex (sex ratio from 1 to 1.2). Men were more hospitalized for acute leukemia, lung, and bladder cancer (sex ratio at 1.5, 3.3, and 5.0, resp.).

Children accounted for half of the patients hospitalized for asthma, one third for respiratory infections and 15% for pneumonia. On the other hand, children accounted for less than 1% of the patients hospitalized for cardiovascular diseases or cancer. Thus, we analyzed these indicators in adults only.

For children, the risk to be hospitalized for respiratory conditions was the same in the high or medium exposed districts and in the reference districts. The risk was slightly increased in districts with low socioeconomic status (Table 5). 

For adults, and for most of the studied indicators, the risk to be hospitalized was the same in areas with medium or high exposure to industrial air pollution and in areas exposed to reference levels. However, the relative risk (RR) to be hospitalized for an acute leukaemia increased significantly to 2.6 for men living in districts with high SO_2_ levels. No increase was observed for women. We found a significant increase of the risk to be hospitalized for myocardial infarction in the districts exposed to industrial air pollution, especially in women (Table 6). 

Excess risk to be hospitalized for MI in women living in districts with medium or high SO_2_ exposure was, respectively, 38% [CI 95% 4% : 83%] and 54% [14% : 110%] greater than women living in districts at the reference level. A 26% [2% : 57%] excess risk to be hospitalized for MI was observed in men living in districts with high SO_2_ levels only compared to those living in districts at reference levels.

## 4. Discussion 

This is the first ecological study on hospitalizations related to industrial air pollution near a large industrial estate in France. It highlights the cardiovascular effects of air pollution. An excess risk of hospitalizations for myocardial infarction was found for women living in the districts exposed to industrial air pollution and for men living in the highly exposed districts. These results are similar to those reported by Fung et al. in a Canadian study, where SHR for cardiovascular and respiratory diseases increased in industrial cities compared to a reference city, with higher ratios in women [5]. On the other hand, a study set in England and Wales did not show any excess risk of hospitalization for cardiovascular, cerebrovascular, and respiratory diseases among the population living near coke works [6]. 

The estimated excess risk of hospitalizations for acute MI was greater in women while men were mostly hospitalized for cardiovascular causes. This could be related to a higher sensitivity of women to the effects of air pollution [33] or to a better control of confounding factors in men than in women. A local study showed a correlation between the socio-occupational group and smoking in men. Daily smoking is twice as much common for workers and unemployed persons than for managers [34]. These differences by socio-occupational group are less pronounced in women. So, the adjustment of the analysis on the proportion of male workers allowed us to control partially smoking in men but not in women.

We did not find excess risk for asthma hospitalizations in children while a case crossover study found a relationship between hospitalizations or emergency visits for asthma attack and SO_2_ peaks in children living near refineries (no association was found when using SO_2_ daily means) [7]. 

The lack of significant results for respiratory diseases most probably shows that hospitalization indicators are not the best indicators to evaluate the respiratory health effects of air pollution in adults in France. Asthma hospitalization rate in adults decreased slightly since 2000, and asthma disease is mostly taken care of by ambulatory management [35]. Studies using emergency or general practitioner (GP) visits for asthma attacks could be more relevant. Moreover most of the published studies concern the analysis of asthma or respiratory symptoms prevalence in children living near industrial sites by comparison to those living in a nonexposed area [36–38]. These studies showed an increase of respiratory symptoms and asthma attacks for exposed children. Pulmonary function tests found a decrease in lung function and an increase of airway inflammation.

Regarding cancer, results reflect past exposure because of the long latency period between exposure and onset of cancer. It would have been much better to estimate patient's exposure 10–15 years ago but we had no information on their place of residence before the hospitalization. Only one significant association was found between the exposure to industrial air pollution and acute leukemia in men. This result must be considered with caution because of the small number of observed cases. However, this association observed in men may suggest a potential occupational exposure due to compounds processed or emitted by petrochemical industries. Some of them are classified as carcinogenic for human (benzene, 1.3-butadiene) or likely carcinogenic for human (1.2-dichloroethane), and benzene is commonly considered as a risk factor for acute myeloid leukemia [39, 40]. This hypothesis needs to be evaluated by local studies on the occupational exposure to these carcinogenic compounds. 

The strength of this study was the estimation of the exposure to industrial air pollution using modeled SO_2_ concentrations rather than a distance to the industrial source. This pollutant was the best proxy of industrial air pollution as industrial sources provided 85% of the total SO_2_ emissions in the study area. Annual mean concentrations of SO_2_ were used in this study rather than hourly values for practical reasons and time consuming. Anyway, monitoring stations with the highest annual means were those with the hourly values too. Using SO_2_ annual mean to model industrial air pollution rather than hourly values should not change the class of exposure of each district.

Particulate matter (PM_10_) concentrations were emitted by many other sources, than industrial sources and could not identify correctly industrial districts. In fact, as shown by the three HRA studies, many pollutants other than SO_2_ are emitted by industries in particular particles. Several studies have shown short-term effects of particulate matter (PM) on hospital admissions from cardiovascular causes [15–22], and myocardial infarctions have been shown to be susceptible to being triggered by PM [16–19]. Population living near industries is exposed to a mixture of pollutants, and particles could play a role in the observed excess of myocardial infarction hospitalizations.

In our study, exposure to air pollution, assessed as the annual average levels of modeled concentrations, depends on the parameters of dispersion and meteorological models. Corrections and adjustments were implemented at each modeling step to limit errors and bias. Using average values for each geographical unit may have resulted in a dilution effect of exposure when modeled concentrations were heterogeneous within districts. We limited this dilution effect by computing the average concentrations only in the urban area, making the hypothesis that people spent most of the time in this area during the day. 

In ecological studies, the choice of exposed and non-exposed areas is usually based on the distance to the industrial site, making the hypothesis that exposure decreases as the distance increases [41–44] whereas a set estimation of exposure would be more relevant. Few studies define the study area with pollutant concentration modeling and GIS. One study used an approach based on SO_2_ and nitrogen dioxides (NO_*x*_) levels, taking only into account levels above limit values. Pollutant levels were interpolated by kriging, and a GIS was used to assign a mean concentration at residential address to each case [45]. Another study used GIS tools to assign an individual integrated score of exposure that accounts for subject's mobility, length of residential stay, distance to petrochemical plants, wind direction, and industrial pollution sources [46]. However, these studies were cross-sectional studies based on individual data and none of them used aggregated health data.

Regarding the design of our study, the main advantage of ecological studies is the use of aggregated data which are often routinely produced, such as hospitalization data. These data are potentially biased by coding or ranking errors that are not differential and lead rather to an underestimation of the relationship with air pollution exposure. The main error of ecological studies is the ecological bias related to heterogeneity in the geographical units due to one or more uncontrolled confounding factors that could be related to exposure and/or to health indicators. 

The socioeconomic status is often seen as a source of heterogeneity between districts. Our models are adjusted on the socioeconomic status estimated by the index of Townsend and the proportion of male workers in the working population. For this local study, the index of Townsend distinguishes relatively well between the industrialized districts and the favored residential municipalities but is more variable in districts under plumes of industries. The highly exposed districts are not always the most deprived districts. For example, Fos-sur-Mer is an industrial highly polluted district but is situated in the middle class for SES.

In the literature, studies carried out on links between social deprivation, health, and air pollution use either several socioeconomic items (average annual income, proportion of people below the poverty threshold, educational level, proportion of unemployed person, proportion of workers, and marital status) or synthetic index of deprivation as those of Townsend [31], Carstairs and Morris [47], or Jarman [48]. Sometimes, synthetic indexes are specifically built from several socioeconomic variables either by a factorial [49, 50] or by an additive approach [51, 52]. These specific indexes, more representative of the local deprivation, are often used to analyze the SES modifying effect on pollution exposure. In our study we used the deprivation index as confounding factor, and Townsend's index seemed to estimate correctly deprivation at district level as reported by Declercq and Prouvost [53].

Determinants of the healthcare system can also potentially modify the relationship between exposure and hospitalizations. In France, access to healthcare is available for the quasi-totality of the population, and the very few access restrictions do not constitute a real limit in our study. On the other hand, the use of health care is linked to the socioeconomic status of the patients [54] and to the socioeconomic context of residence [55]. We did not control directly the possible heterogeneity in the use of health care because of the lack of available data at district level. However, it was indirectly taken into account through the index of Townsend and by the Bayesian hierarchical model controlling the spatial autocorrelation. This modeling allowed us to limit the bias due to variability in use of health care between districts. 

Finally, in the ecological studies, the individual confounding factors such as obesity, cholesterol level, lifestyle, smoking, and alcoholism cannot be taken into account because of using aggregated data at district level. 

## 5. Conclusion

This study underlines that, in terms of hospitalizations for respiratory diseases and cancers, the health condition of the population exposed to the industrial air pollution was similar to those of nonexposed people. However, the results illustrate the impact of industrial air pollution on the cardiovascular system. 

Efforts should be done to decrease the levels of SO_2_, particles, and some carcinogenic compounds emitted by the industries, by improving industrial processes and using less polluted fuels. For instance, decreasing the level of road traffic particles would require the implementation of an interurban public transport network, as well as the development of rail transport for raw materials and goods.

Prevention of the cardiovascular diseases should be a public health priority in the study area, particularly in women. General practitioners, key players in the health prevention, would have clear and useful information on harmful cardiovascular effects of air pollution.

Finally, occupational medicine should reinforce the screening of hematopoietic disorders, myelodysplasia, and acute leukaemia in workers as well as in pensioners of refineries and petrochemical plants.

## Figures and Tables

**Table 1:** Annual mean, maximum daily mean, and maximum hourly mean of SO_2_ concentrations (*µ*g·m^−3^) measured by monitoring stations located in the study area and in the remaining part of the regional area (2008 data).

Monitoring station	Type	Annual mean	Maximum daily mean	Maximum hourly mean
Study area				

Berre-l'Etang	Urban	7	66	190
Berre Magasin	Urban	4	29	160
Carry-le-Rouet	Industrial	6	41	200
Chateauneuf/La Mède	Industrial	5	74	404
Chateauneuf les Martigues	Industrial	4	28	124
Fos-sur-Mer	Urban	15	138	427
Fos-sur-Mer/les Carabins	Urban	2	33	200
Istres	Urban	5	33	125
La Fare les Oliviers	Industrial	5	22	122
Marignane ville	Urban	NA	32	221
Martigues l'île	Urban	7	43	327
Martigues La Couronne	Industrial	8	79	407
Martigues La Gatasse	Industrial	10	121	759
Martigues Lavéra	Industrial	9	88	522
Martigues Les Laurons	Industrial	18	151	412
Martigues Les Ventrons	Industrial	10	134	831
Martigues NDM	Urban	4	71	380
Martigues Le Pati	Industrial	6	36	230
Miramas ville	Urban	6	23	115
Port de Bouc La lèque	Urban	15	126	375
Port de Bouc Castillon	Industrial	11	73	292
Port de Bouc EDF	Urban	10	70	274
Port Saint Louis	Industrial	4	27	134
Rognac les Barjaquets	Industrial	4	69	350
Salon-de-Provence	Urban	4	19	83
Sausset les Pins	Industrial	10	79	433
Vitrolles	Urban	7	39	164

Remaining part of the regional area				

Arles	Urban	3	12	58
Les Pennes-Mirabeau	Urban	3	24	132
Marseille Cinq-Avenues	Urban	4	25	125
Nice Pellos	Traffic	4	18	43
Peillon	Industrial	4	10	22
Contes	Industrial	1	5	20

**Table 2:** Distribution of estimated SO_2_ and PM_10_ concentrations by district (2008 data).

Pollutant indicator	Mean	Minimum	Centile20	Centile40	Centile60	Centile80	Maximum
PM_10_	29.8	27.9	28.8	29.3	30.8	32.2	33.6
SO_2_	4.4	2.1	3.4	4.2	4.6	6.4	12.4

**Table 3:** Annual mean, maximum daily mean, and maximum hourly mean of PM_10_ concentrations (*µ*g·m^−3^) measured by monitoring stations located in the study area and in the remaining part of the regional area (2008 data).

Monitoring station	Type	Annual mean	Maximum daily mean
Study area			

Chateauneuf/La Mède	Industrial	32	102
Fos-sur-Mer/les Carabins	Urban	31	93
Marignane ville	Urban	33	106
Martigues l'île	Urban	27	84
Miramas ville	Urban	28	86
Port de Bouc La lèque	Urban	32	87
Port Saint Louis	Industrial	29	82
Rognac les Barjaquets	Industrial	27	93
Salon-de-Provence	Urban	31	94

Remaining part of the regional area			

Arles	Urban	29	90
Marseille Cinq-Avenues	Urban	29	87
Marseille Saint Louis	Urban	31	82
Marseille Thiers	Urban	27	76
Marseille Timone	Traffic	33	88
Aix Ecole d'Art	Urban	28	83
Aix jas de Bouffan	Urban	27	82
Aix Roy René	Traffic	32	86
Gardanne	Industrial	37	101
Hyères	Urban	26	74
Toulon Chalucet	Urban	28	80
Toulon foch	Traffic	38	131
Avignon Mairie	Urban	25	80
Le Pontet	Urban	31	309
Antibes Jean Moulin	Suburban	34	71
Cannes Broussailles	Urban	35	69
Nice aéroport	Observation	34	74
Cagnes sur Mer	Urban	31	74
Contes	Industrial	43	100
Peillon	Industrial	39	105

**Table 4:** Number of cases and distribution by quartiles for each hospitalization indicator between 2004 and 2007.

Hospitalization indicators	Cases	Min	Q25	Q50	Q75	Max
All cardiovascular diseases	26,108	188	397	585	1,319	3,002
Heart diseases	14,506	90	200	315	752	1,729
Coronary heart diseases	4,684	29	71	105	258	577
Coronary heart diseases with heart rate disorders	808	0	13	17	41	99
Myocardial infarction	1,545	8	19	37	93	223
Stroke	4,008	19	57	89	230	553
Heart rate disorders	2,026	10	26	47	125	267

All respiratory diseases	16,107	117	188	317	872	1,823
Respiratory infections	4,574	21	51	92	287	664
Pneumonia	2,839	15	34	57	183	394
Asthma	937	2	9	17	54	131
Exacerbation of chronic obstructive pulmonary disease (COPD)	1,213	3	13	24	69	160

All cancers	10,416	89	159	249	499	1,251
Breast cancer	1,441	14	24	34	53	183
Lung cancer	879	3	13	21	50	119
Bladder cancer	515	1	8	14	26	68
Malignant non-Hodgkin's lymphoma	311	1	5	7	15	36
Acute leukemia	138	0	2	4	7	15
Myeloma	121	0	1	2	6	18

**Table 5:** RR of respiratory hospitalizations and 95% credible interval (CI) in children.

Hospitalizations indicators	Exposure class	RR	IC 95%
	Reference	1	
All respiratory diseases	Medium	0.93	[0.77–1.15]
	High	0.86	[0.68–1.10]

	Reference	1	
Respiratory infections	Medium	**0.69**	**[0.53**–**0.90]**
	High	0.79	[0.59–1.06]

	Reference	1	
Pneumonia	Medium	0.67	[0.41–1.17]
	High	0.70	[0.38–1.36]

	Reference	1	
Asthma	Medium	0.71	[0.49–1.04]
	High	0.84	[0.54–1.35]

**Table 6:** RR of cardiovascular, respiratory and cancer hospitalizations and 95% credible interval (CI) in adults.

Hospitalizations indicators	Exposure class	Males		Females	
		RR	CI 95%	RR	CI 95%
All cardiovascular diseases	Reference	1		1	
	Medium	1.03	[0.95–1.11]	1.01	[0.90–1.12]
	High	0.96	[0.88–1.05]	0.91	[0.80–1.04]
Heart diseases	Reference	1		1	
	Medium	1.08	[0.97–1.19]	1.13	[0.96–1.32]
	High	0.98	[0.87–1.11]	0.99	[0.82–1.19]
Coronary heart diseases	Reference	1		1	
	Medium	1.13	[0.93–1.36]	1.22	[0.87–1.65]
	High	1.07	[0.86–1.34]	1.11	[0.76–1.61]
Myocardial infarction	Reference	1		1	
	Medium	1.13	[0.94–1.37]	**1.38**	**[1.04–1.83]**
	High	**1.26**	**[1.02–1.57]**	**1.54**	**[1.14–2.10]**
Heart rate disorders	Reference	1		1	
	Medium	1.15	[0.98–1.35]	1.01	[0.81–1.31]
	High	1.16	[0.98–1.40]	1.05	[0.80–1.40]
Coronary heart diseases with heart rate disorders	Reference	1		1	
	Medium	0.94	[0.73–1.20]	1.06	[0.76–1.47]
	High	1.05	[0.73–1.52]	0.90	[0.61–1.31]
Stroke	Reference	1		1	
	Medium	0.97	[0.80–1.19]	1.07	[0.82–1.37]
	High	1.07	[0.86–1.34]	0.86	[0.60–1.15]

All respiratory diseases	Reference	1		1	
	Medium	0.97	[0.84–1.10]	1.08	[0.90–1.27]
	High	1.01	[0.86–1.20]	1.05	(0.86–1.29]
Respiratory infections	Reference	1		1	
	Medium	0.87	[0.68–1.11]	0.85	[0.65–1.09]
	High	1.00	[0.76–1.32]	0.97	[0.73–1.30]
Pneumonia	Reference	1		1	
	Medium	0.87	[0.68–1.13]	0.80	[0.63–1.03]
	High	0.95	[0.72–1.29]	0.93	[0.69–1.24]
Acute COPD	Reference	1		1	
	Medium	0.87	[0.57–1.31]	1.16	[0.77–1.80]
	High	0.80	[0.49–1.32]	0.97	(0.59–1.64]

All cancers	Reference	1		1	
	Medium	0.98	[0.89–1.07]	1.00	[0.91–1.11]
	High	0.90	[0.81–1.01]	1.03	[0.92–1.15]
Lung cancer	Reference	1		1	
	Medium	1.02	[0.81–1.29]	0.79	[0.51–1.28]
	High	1.09	[0.84–1.43]	1.05	[0.65–1.78]
Breast cancer	Reference	na	na	1	
	Medium	na	na	1.07	[0.91–1.25]
	High	na	na	0.99	[0.82–1.20]
Bladder cancer	Reference	1		1	
	Medium	0.83	[0.56–1.26]	1.07	[0.58–2.12]
	High	0.76	[0.48–1.23]	0.81	[0.38–1.70]
Acute leukemia	Reference	1		1	
	Medium	1.86	[0.87–4.13]	1.08	[0.38–3.97]
	High	**2.57**	**[1.10–6.33]**	0.94	[0.29–4.24]
Myeloma	Reference	1		1	
	Medium	1.35	[0.54–3.61]	0.73	[0.32–1.62]
	High	1.65	[0.57–5.07]	0.40	[0.15–1.02]
Malignant non-Hodgkin's lymphoma	Reference	1		1	
	Medium	0.81	[0.52–1.23]	0.88	[0.51–1.59]
	High	0.61	[0.35–1.00]	0.86	[0.47–1.63]

Na: not available.
